# *In vitro* DNA/RNA Adductomics to Confirm DNA Damage Caused by Benzo[*a*]pyrene in the Hep G2 Cell Line

**DOI:** 10.3389/fchem.2019.00491

**Published:** 2019-07-09

**Authors:** Toshihide Takeshita, Robert A. Kanaly

**Affiliations:** Department of Life and Environmental System Science, Graduate School of Nanobiosciences, Yokohama City University, Yokohama, Japan

**Keywords:** DNA adducts, RNA adducts, Hep G2, DNA adductomics, RNA adductomics

## Abstract

In the development of new chemical substances, genetic toxicity evaluations are a high priority for safety risk management. Evaluation of the possibility of compound carcinogenicity with accuracy and at reasonable cost in the early stages of development by *in vitro* techniques is preferred. Currently, DNA damage-related *in vitro* genotoxicity tests are widely-used screening tools after which next generation toxicity testing may be applied to confirm DNA damage. DNA adductomics may be used to evaluate DNA damage *in vitro*, however confirmation of DNA adduct identities through comparison to authentic standards may be time-consuming and expensive processes. Considering this, a streamlined method for confirming putative DNA adducts that are detected by DNA adductomics may be useful. With this aim, *in vitro* DNA adductome methods in conjunction with *in vitro* RNA adductome methods may be proposed as a DNA adductome verification approach by which to eliminate false positive annotations. Such an approach was evaluated by conducting *in vitro* assays whereby Hep G2 cell lines that were exposed to or not exposed to benzo[*a*]pyrene were digested to their respective 2'-deoxynucleosides or ribonucleosides and analyzed by liquid chromatography electrospray ionization tandem mass spectrometry (LC/ESI-MS/MS) by comparative DNA and RNA adductomics through neutral loss targeting of the [M + H]^+^ > [M + H – 116]^+^ or [M + H]^+^ > [M + H −132]^+^ transitions over predetermined ranges. Comparisons of DNA adductome maps revealed putative DNA adducts that were detected in exposed cells but not in unexposed cells. Similarly, comparisons of RNA adductome maps revealed putative RNA adducts in exposed cells but not in unexposed cells. Taken together these experiments revealed that analogous forms of putative damage had occurred in both DNA and RNA which supported that putative DNA adducts detected by DNA adductomics were DNA adducts. High resolution mass spectrometry (HRMS) was utilized to confirm that putative nucleic acid adducts detected in both DNA and RNA were derived from benzo[*a*]pyrene exposure and these putative adducts were identified as 7,8-dihydroxy-9,10-epoxy-7,8,9,10-tetrahydrobenzo[*a*]pyrene- (B[*a*]PDE)-type adducts. Overall, this study demonstrates the usefulness of utilizing DNA/RNA adductomics to screen for nucleic acid damage.

## Introduction

Genetic toxicity evaluation of new chemicals is a high priority in safety risk management and evaluations that focus on whether a new chemical may induce mutagenicity and/or carcinogenicity are required as part of hazard identification and risk characterization (Cimino, 2006; Petkov et al., 2015; Thybaud et al., 2017). Assaying for tumor formation after chemical administration to animals *in vivo* may be utilized for evaluating carcinogenicity, however high cost, long assay times and issues related to animal protection must be considered (Bourcier et al., 2015; Petkov et al., 2015). *In vitro* methods to evaluate genotoxicity at the early stages of chemical product development include assays such as the Ames test, micronucleus test, and the chromosomal aberration test, which taken together may be utilized to predict the carcinogenic potential of a chemical (Kirkland et al., 2005; Hayashi et al., 2013). Currently, improved *in vitro* testing methods that may include the evaluation of DNA damage for predicting mutagenicity and carcinogenicity of chemical compounds with accuracy and at sufficiently low cost in the early stages of chemical development are sought (MacGregor et al., 2015; Petkov et al., 2015; Dertinger et al., in press).

One of the fundamental mechanisms in the initiation of events that may lead to carcinogenesis is damage to DNA (Poirier, 2004). DNA damage in the form of bulky DNA adducts may be derived from direct reactions of chemicals with DNA or following bioactivation (Koreeda et al., 1978). In turn, bulky DNA adducts may cause increases in the frequency of mutations by triggering translesional DNA synthesis repair mechanisms that increase the risks of point mutations thus continuing the process toward carcinogenesis (Zhang et al., 2000). Performing direct molecular level detection of chemical modifications to DNA in living cells contributes to our understanding of the potential causes of initiation of carcinogenesis and provides valuable information toward interpreting genotoxicity test results that may clarify modes of action mechanisms (Preston and Williams, 2005; Dertinger et al., in press). At the same time, high rates of false positive test results in *in vitro* assays are a challenging issue and suggest the need for an increased focus on mechanistic (i.e., pathway-based) understandings of toxicity at the early stages of testing (Kirkland et al., 2005, 2007; Honda et al., 2018; Sobus et al., 2018).

DNA adductomics may have potential as a candidate methodology as part of DNA damage/genotoxicity screening because it takes a comprehensive approach to investigating DNA damage through direct molecular level detection of chemical modifications for the purposes of identification and quantification of DNA adducts (Kanaly et al., 2006, 2007; Balbo et al., 2014; Hemeryck et al., 2017; Chang et al., 2018; Dertinger et al., in press). For example, applications of DNA adductomics to assess DNA damage of compounds that tested positive in the micronucleus test were conducted and it was proposed that DNA adductomics may be useful in the determination of false-positive compounds from this test (Kato et al., 2011). At the same time, however, although DNA adductomics may have potential to become a useful technique to evaluate DNA damage/genotoxicity *in vitro*, confirmation of DNA adduct identities through DNA adduct compound syntheses is a time-consuming and expensive process. Considering this, alternative methods to circumvent these issues are needed if DNA adductomics is to be applied to *in vitro* testing.

In this research, a methodology which may help to streamline DNA adductomics analyses in *in vitro* testing is proposed. The methodology is an *in vitro* comparative DNA/RNA adductomics method that was applied to a model human hepatoma cell line, Hep G2 for the purpose of confirming DNA adducts detected by DNA adductomics without the need to synthesize DNA adduct standards.

## Materials and Methods

### Biochemical and Chemical Reagents

Bovine spleen phosphodiesterase II (SPD) and micrococcal nuclease (MN) were purchased from Worthington Biochemical Corp. (Lakewood, NJ, USA). Bacterial alkaline phosphatase Type III (*E. coli*), 2′-deoxycytidine, 2′-deoxythymidine, 2′-deoxyadenosine, 2′-deoxyguanosine and 2′,3′-dideoxyinosine were purchased from Sigma-Aldrich Co. (St. Louis, MO, USA). Benzo[*a*]pyrene (>98%), adenosine (>98%), phosphate buffered saline(-) [PBS(-)], methanol (LC/MS grade), and dimethyl sulfoxide (DMSO) were purchased from Wako Chemical (Osaka, Japan). Guanosine (>98%), cytidine (>98%) and uridine (>98%) were purchased from Tokyo Chemical Industry Co (Tokyo, Japan).

### Cell Culture

The human hepatoma cell line, Hep G2, was obtained from the Japanese Collection of Research Bioresources Cell Bank (JCRB, Osaka, Japan). Cells were maintained in 10 ml of low glucose Dulbecco's minimal essential medium supplemented with fetal bovine serum (DMEM-FBS, 5.0%; Wako Chemical), penicillin, 100 U/ml, and streptomycin, 100 μg/ml (Wako Chemical, 168-23191). Stock cultures were grown at 37°C in an atmosphere of 5% CO_2_ and 95% relative humidity.

### Growth and Exposure of Hep G2 Cells to Benzo[*a*]pyrene

Hep G2 cells were grown in culture medium as described above in 100 mm diameter size tissue culture dishes until confluent monolayers were obtained. Exposure and incubation of Hep G2 cells was conducted as described previously (Takeshita et al., 2019). Briefly, in this work, benzo[*a*]pyrene, 10 μM concentration in medium, was administered to cells in DMSO (10 μl, 0.1% v/v) whereby benzo[*a*]pyrene in DMSO was sterilized by filtration through Millex-LG 0.22 μm PTFE filters (Merck, Kenilworth, NJ, USA). Unexposed cells were treated with equal amounts of filter-sterilized DMSO without benzo[*a*]pyrene. Exposed and unexposed Hep G2 cells were incubated separately under identical conditions at 37°C in an atmosphere of 5% CO_2_ and 95% relative humidity. After 24 h, the culture medium was removed by aspiration and the remaining cells were gently washed two times with 3 ml of PBS(-) in their respective culture dishes. PBS(-) was removed by aspiration each time and then 1 ml of 0.25% w/v trypsin-1 mmol/l EDTA·4Na solution with phenol red (Wako, Japan) was added to each dish. Cells in their respective culture dishes were then placed in a 37°C incubator for 3 min. After 3 min, cells in trypsin were suspended in 5 ml of DMEM-FBS, contents were transferred by glass pipette to 15-ml size tubes (FALCON) and then centrifuged at 500 × g for 3 min. Finally, all supernatant was removed by aspiration and pelleted cells were stored at −80°C before DNA or RNA extraction.

### DNA Extraction and Enzymatic Digestion

Total DNA was extracted from Hep G2 cells by using the Quick-DNA Universal Kit (Zymo Research, Irvine, CA, USA) according to the manufacturer's instructions. Afterwards DNA quantification and purity were measured by absorbance at 260 and 280 nm via a NanoVue UV-Visible Spectrophotometer (General Electric Healthcare Life Sciences, Pittsburgh, PA, USA). Based upon the amount of DNA recovered from each sample (between approximately 30 and 100 μg per treatment condition), aliquots of elution buffer that contained DNA were transferred to 1.5-ml size tubes and buffer was removed by vacuum centrifugation (Tomy Seiko Co., Tokyo, Japan) and stored at −80°C.

DNA was solubilized in mixed buffer (17 mM sodium succinate and 8 mM calcium chloride, pH 6.0) and hydrolyzed enzymatically to 2′-deoxyribonucleoside-3′-monophosphates utilizing 67.5 units of micrococcal nuclease and 0.225 units of spleen phosphodiesterase in a total volume of 54 μl. Solutions were incubated for 3 h at 37°C in a heating block (DTU-1B, Taitec, Saitama, Japan). Next, digestion to obtain the corresponding 2′-deoxyribonucleosides was conducted by incubating the samples in 245 μl-volume solution of 60 mM Tris-HCl (pH 8.5), 1.2 mM zinc sulfate and 9 units of alkaline phosphatase for 3 h at 37°C. The digested samples were subjected to vacuum centrifugation and extracted twice with methanol. The methanol fraction was removed by vacuum centrifugation, and 2′-deoxynucleosides were stored at −80°C. Before LC/ESI-MS/MS analysis, samples were adjusted to represent equal amounts of 2′-deoxynucleosides in a of 20% (w/v) mixture of DMSO in Milli-Q water that contained 2′,3′-dideoxynosine as an calibration standard.

### RNA Extraction and Enzymatic Digestion

Total RNA was extracted from Hep G2 cells by using a NucleoSpin®RNA Kit (Macherey-Nagel, Düren, Germany) according to the manufacturer's instructions and RNA quantification and purity were confirmed as described above. Extracted RNA was solubilized in mixed buffer and digested to their corresponding ribonucleosides also as described above however after the first digestion, 245 μl of a solution that contained 60 mM Tris-HCl (pH 8.5), 1.2 mM zinc sulfate, 10 units of RNase A, 2 units of nuclease P1, and 9 units of alkaline phosphatase was added and digestion was continued for 3 h at 37°C. Digested RNA samples were subjected to vacuum centrifugation to remove buffer and methanol extraction after which methanol was also removed by vacuum centrifugation as per the DNA extraction procedure. Finally, samples were adjusted to represent equal amounts of nucleosides in a of 20% (w/v) mixture of DMSO in Milli-Q water that contained 2′,3′-dideoxynosine as a calibration standard.

### DNA and RNA Adductome Mapping by LC/ESI-MS/MS

Analyses of 2′-deoxynucleosides and ribonucleosides were conducted by liquid chromatography electrospray ionization-tandem mass spectrometry in positive ionization mode (LC/ESI(+)-MS/MS). The LC system consisted of a Waters model 2690 Separations Module that was in-line with a Waters model 2998 photodiode array detector (Waters Corp., Milford, MA, USA) and it was interfaced with a Quattro *Ultima* triple stage quadrupole mass spectrometer (Waters-Micromass, Manchester, UK). Generally, an aliquot of sample, 20 μl, was injected via autosampler onto an XSelect CSH C18 column (4.6 mm i.d. × 150 mm, 3.5 μm particle size, Waters) that was in-line with a Security Guard Cartridge System pre-column fitted with a widepore C18 cartridge (Phenomenex, Torrance, CA, USA). Samples were eluted at a flow rate of 0.2 ml/min utilizing gradient mode. Starting conditions were 5% methanol and 95% water and changed to 80% methanol and 20% water over a period of 25 min. These conditions were held for 5 min at a flow rate of 0.3 ml/min and then returned to the original starting conditions through 60 min at a flow rate of 0.3 ml/min. Mobile phase was transferred to the mass spectrometer by electrospray which utilized nitrogen gas as the nebulizing gas. The ion source temperature was 130°C, the desolvation temperature was 350°C, and the cone voltage was 35 V. Nitrogen gas was used as the desolvation gas, 600 L/h, and cone gas, 60 L/h. Argon gas (99.9999%) was used as the collision cell gas at a collision cell pressure of approximately 4 × 10^−3^ mbar and the collision cell energy was 10 eV for DNA (Takeshita et al., 2019) and RNA (unpublished data) adductome analyses. The MS was operated in multiple reaction monitoring mode (MRM) utilizing positive ionization. Analyses of DNA samples were conducted by monitoring the neutral loss of 2′-deoxyribose from positively ionized 2′-deoxynucleoside adducts corresponding to [M + H]^+^ > [M + H – 116]^+^. Analyses of RNA samples were conducted by monitoring the neutral loss of ribose from positively ionized ribonucleoside adducts corresponding to [M + H]^+^ > [M + H – 132]^+^. Analyses of authentic standard mixtures of 2′-deoxynucelosides (2′-deoxycytidine, 2′-deoxythymidine, 2′-deoxyadenosine, 2′-deoxyguanosine) and ribonucleosides (cytidine, uridine, adenosine, guanosine) were conducted routinely to confirm LC conditions and ionization efficiency based upon retention times and signal strengths. One transition in each sample run was reserved to monitor the internal standard, 2′,3′-dideoxyinosine at transition 236.9 > 136.8. 2′-Deoxynucleosides and ribonucleosides were monitored by UV detection at 254 nm. Data from LC/ESI(+)-MS/MS analyses were processed by using MassLynx software whereby a peak area threshold of 1,000 arbitrary units was utilized. Peak areas were normalized based upon (1) the amounts of 2′-deoxyguanosie (DNA samples) or guanosine (RNA samples) detected by UV (Supplemental Material) and (2) the relative ionization efficiency for each sample run as detected by the signal intensity of 2′,3′-dideoxyinosine. Finally, DNA and RNA adductome mapping was conducted as described previously (Kanaly et al., 2015).

### Confirmation of Benzo[*a*]pyrene Exposure-Derived DNA and RNA Adducts by LC -HRMS

Chromatographic separation and high resolution mass analyses of putative DNA and RNA adducts were conducted by using a Thermo Scientific Ultimate 3000 HPLC coupled to a Thermo Scientific model Q Exactive Focus hybrid quadrupole-Orbitrap mass spectrometer. Analysis methods were developed based upon the LC/ESI-MS/MS conditions utilized above and Villalta et al. (2017). An aliquot of sample, 20 μl, was injected via autosampler onto an XSelect CSH C18 column (4.6 mm i.d. × 150 mm, 3.5 μm particle size, Waters) that was in-line with a Security Guard Cartridge System pre-column fitted with a widepore C18 cartridge (Phenomenex, Torrance, CA, USA). The flow rate and gradient conditions were as described above except that flow was diverted to waste from 0 to 10 min and from 45 to 60 min. Based upon the DNA and RNA adductomics analyses, the parent masses of the putative adducts were targeted for HRMS MS2 analysis at a resolving power of 70,000. The HRMS was operated using a heated electrospray ionization source (HESI) in positive electrospray ionization mode using a parallel reaction monitoring (PRM) experiment to monitor MS^2^ spectra obtained by CID of the protonated target molecules [M + H]^+^. The precursor ions filtered by the quadrupole in a 0.4 *m/z* isolation window were fragmented in the higher-energy collisional dissociation (HCD) collision cell utilizing stepped collision energies of 10 and 15 eV. Product ions were detected in the mass analyzer at an automatic gain control (AGC) target value of 5 × 10^4^ and a maximum injection time (IT) that was dependent upon the highest resolution of the active and previous scan event. The HESI probe parameters were set whereby the flow rates of the sheath gas, auxiliary gas and sweep gas (all nitrogen) were: 45 arbitrary units, 10 arbitrary units, and 2 arbitrary units, respectively. The probe heater was 400°C, the capillary temperature was 250°C, the ion spray voltage was 3.5 kV and the S-lens RF level was 50. Thermo Scientific Xcalibur software was used for data acquisition and processing.

## Results

### Comparative DNA and RNA Adductome Analyses

**Figure 1:**
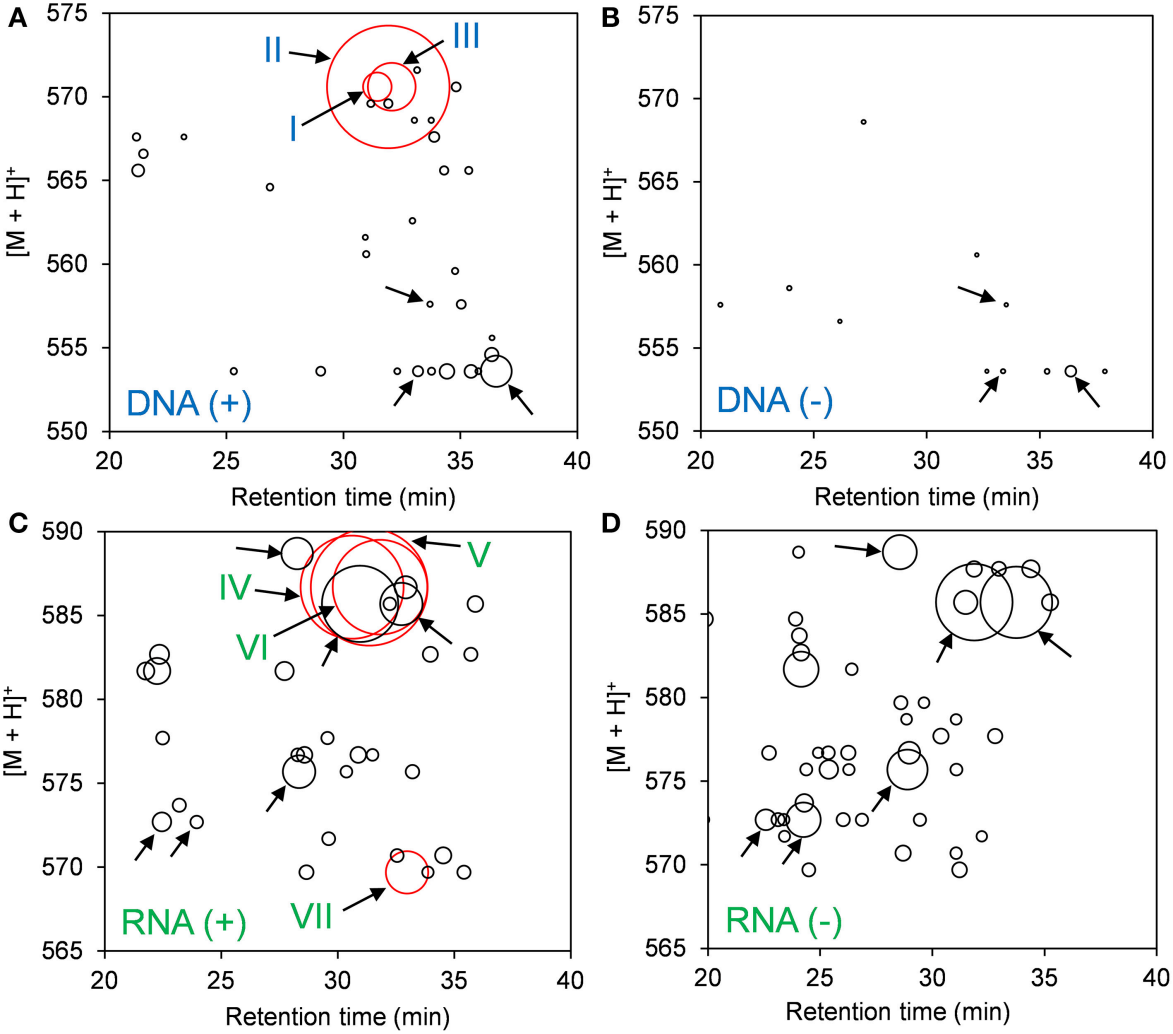
Results of comparative DNA/RNA adductomics analyses. DNA or RNA was extracted from Hep G2 cells that were exposed or not exposed to benzo[*a*]pyrene, digested to 2′-deoxynucelosides or ribonucleosides, purified and analyzed by LC/ESI(+)-MS/MS utilizing SRM mode. **(A)** Exposed cells, DNA; **(B)** unexposed cells, DNA; **(C)** exposed cells, RNA; and **(D)** unexposed cells, RNA. Putative DNA adducts discussed in the text are represented by red bubbles and arrows are labeled I through VII. Unlabeled arrows draw attention to putative adducts that were detected identically in exposed and unexposed cells from DNA **(A,B)** and RNA **(C,D)**.

DNA adductome and RNA adductome maps were constructed separately for Hep G2 cells after 24 h of exposure to benzo[*a*]pyrene and after 24 h under the same conditions but without exposure as described in the Materials and Methods (Figure 1). DNA adductome mapping showed that putative DNA adducts were detected in both exposed and unexposed cells over the corresponding ranges (Figures 1A,B) and that some putative DNA adducts occurred at similar retention times and relative abundances in both samples (indicated by unlabeled arrows). Comparison of DNA adductome maps from exposed and unexposed cells also revealed three relatively abundant putative DNA adducts that eluted between 30 and 35 min that were detected in cells that were exposed to benzo[*a*]pyrene but that were not detected in unexposed cells (Figures 1A,B). These three putative adducts, labeled I through III in Figure 1A corresponded to identical protonated molecules, [M + H]^+^ = 570, however their retention times appeared to be different.

The results of RNA adductome mapping are shown in Figures 1C,D. Mapping of RNA digests also revealed numerous putative RNA adducts in exposed and unexposed cells that were detected in both samples over the corresponding range. Again it was revealed that many of the putative RNA adducts occurred at similar retention times and similar relative abundances (unlabeled arrows). In many cases, putative RNA adducts appeared under one condition that did not appear in the other. Overall more putative RNA adducts were detected in unexposed cells. At the same time, the three most abundantly-detected putative RNA adducts all occurred in exposed cells between approximately 28 and 34 min (Figures 1C,D). These putative RNA adducts corresponded to identical protonated molecules, [M + H]^+^ = 586, albeit with different retention times. A fourth relatively abundantly-detected putative RNA adduct was detected in exposed cells that was not detected in unexposed cells and it eluted at approximately 33 min and corresponded to [M + H]^+^ = 570 (Figure 1C).

**Figure 2:**
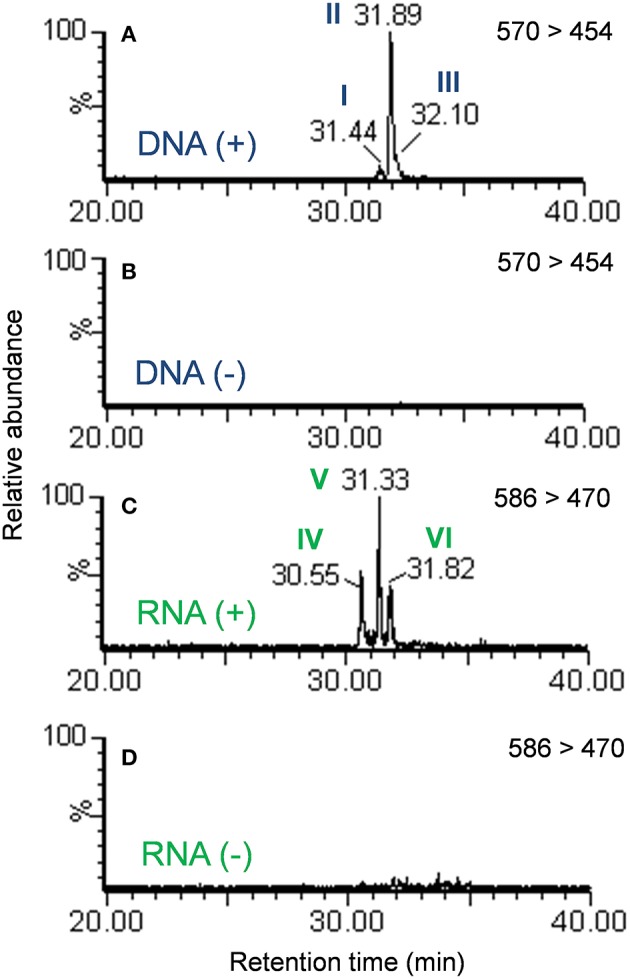
Extracted ion chromatograms of the mass transition equal to 570 > 454 from the DNA of cells exposed to benzo[*a*]pyrene **(A)** or not exposed to benzo[*a*]pyrene **(B)**. Extracted ion chromatograms of the mass transition equal to 586 > 470 from the RNA of cells exposed to benzo[*a*]pyrene **(C)** or not exposed to benzo[*a*]pyrene **(D)**. Peaks are labeled in accordance with Figure 1.

Examination of the extracted ion chromatograms (EICs) of transitions that corresponded to [M + H]^+^ = 570 > 454 in DNA for exposed and unexposed cells showed that putative DNA adducts I through III eluted at retention times of approximately 31.4, 31.9, and 32.1 min, respectively when compared to unexposed cell controls (Figures 2A,B). At the same time, putative RNA adducts IV through VI, [M + H]^+^ = 586 > 470, eluted at retention times of approximately 30.6, 31.3, and 31.8 min, respectively and were not detected in unexposed cells when compared to unexposed cell controls (Figures 2C,D). The presence of these adducts was confirmed by analyses of triplicate experiments and the standard deviations for recovery of each adduct ranged from 5.4 to 12.7% for DNA adducts I through III (*n* = 3 each) and 17.1–20.5 % for RNA adducts IV through VI (*n* = 3 each).

### Confirmation of Benzo[*a*]pyrene Exposure-Derived DNA and RNA Adducts

**Figure 3:**
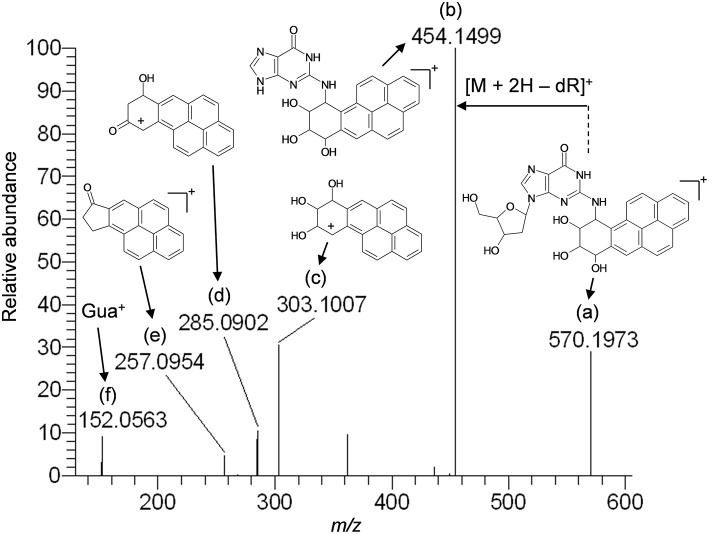
Results of LC-HRMS product ion scan analyses of putative DNA adduct II which corresponded to the protonated molecule [M + H]^+^ = 570.1973. It was targeted for product ion scan analyses after it was revealed by comparative DNA adductome analyses shown in Figure 1A. dR, 2′-deoxyribose; Gua^+^, protonated guanine. **(a–f)** mass fragments.

Based upon the results of untargeted DNA and RNA adductomics analyses, targeted LC-HRMS MS^2^ experiments were conducted to confirm whether the analytes detected in exposed cells were DNA adducts or RNA adducts, and if so, if they were related to benzo[*a*]pyrene exposure. The results of LC-HRMS CID analyses of the most abundant putative DNA adduct, II, is shown in Figure 3. Five abundant diagnostic product ions were detected at *m/z* 454.1499, *m/z* 303.1007, *m/z* 285.0902, *m/z* 257.0954, and *m/z* 152.0563. Detection of *m/z* 152.0563 provided strong evidence that putative adduct II was derived from a 2′-deoxyguanosine (dGuo) adduct because this fragment represented protonated guanine (Gua^+^, Figure 3). Detection of *m/z* 454.1499 provided confirmatory evidence for the loss of 2′-deoxyribose from the parent protonated molecule ([M + 2H – dR]^+^) and corresponded to a loss of 116.0474 amu. The fragment that corresponded to *m/z* 303.1007 represented the positively charged 7,8,9,10-tetrahydrobenzo[*a*]pyrene-7,8,9-triol moiety as shown in Figure 3. Indeed, product ions *m/z* 285.0902 and *m/z* 257.0954 represented losses of water and losses of water plus a carbonyl from the benzo[*a*]pyrene-7,8,9-triol, respectively (Figure 3). Considering these results and a molecular formula of C_30_H_27_N_5_O_7_, an adduct that was formed through the reaction of 2′-deoxyguanosine in DNA and a benzo[*a*]pyrene biotransformation product was proposed as shown in Figure 3. At the same time, comparison of the observed mass value for the protonated molecule of putative adduct II, [M + H]^+^ = 570.1973 and the theoretical mass value for the proposed adduct structure of putative adduct II, [M + H]^+^ = 570.1983, revealed a mass error of 1.8 ppm (Table 1). These results supported further that the identity of this putative adduct was a 7,8-dihydroxy-9,10-epoxy-7,8,9,10-tetrahydrobenzo[*a*]pyrene-2′-deoxyguanosine (B[*a*]PDE-dGuo) diastereomer formed through biotransformation of benzo[*a*]pyrene by Hep G2 cells. Similarly, analyses of the observed mass values and theoretical mass values for all of the CID fragmentation products in accordance with their proposed structures (Table 1 and Figure 3) showed that all had occurred at mass errors <3 ppm each which provided further confirmation for the proposed fragment structures. The mass fragments detected in this work are also in close agreement with mass fragments detected by positive ionization mode CID analyses of B[*a*]PDE-dGuo adducts conducted by Ruan et al. (2006).

**Table 1:** Results of LC-HRMS CID analyses of putative DNA adduct II.

**Peak designation^a^**	**Observed mass fragment value, *m/z***	**Theoretical mass fragment value, *m/z***	**Mass error (ppm)**	**Molecular formula**
^a^	570.1973	570.1983	1.8	C_30_H_28_N_5_$O_{7}{}^{+}$
b	454.1499	454.1510	2.4	C_25_H_20_N_5_$O_{4}{}^{+}$
c	303.1007	303.1016	2.9	C_20_H_15_$O_{3}{}^{+}$
d	285.0902	285.0910	2.8	C_20_H_13_$O_{2}{}^{+}$
e	257.0954	257.0961	2.7	C_19_H_13_O^+^
f	152.0563	152.0567	2.6	C_5_H_6_N_5_O^+^

a *Peak designations correspond to Figure 3*.

The observed masses for putative DNA adducts I and III as shown in Table 2, provided strong evidence that these compounds were also diastereomers of B[*a*]PDE-dGuo (mass errors <1.5 ppm each).

**Table 2:** Summary of results of DNA and RNA LC/ESI-MS/MS adductomics and confirmatory LC-HRMS analyses.

**Nucleic acid adduct^^a^^**	**Adductome [M + H]^**+**^**	**Retention time (min)**	**Observed m/z^^b^^**	**Theoretical m/z^^b^^**	**Mass error (ppm)**	**Identity^^c^^**
I (DNA)	570	31.4	570.1975	570.1983	1.4	B[*a*]PDE-dGuo I
II (DNA)	570	31.9	570.1973	570.1983	1.8	B[*a*]PDE-dGuo II
III (DNA)	570	32.1	570.1978	570.1983	0.9	B[*a*]PDE-dGuo III
IV (RNA)	586	30.6	586.1925	586.1932	1.3	B[*a*]PDE-Guo I
V (RNA)	586	31.3	586.1920	586.1932	2.1	B[*a*]PDE-Guo II
VI (RNA)	586	31.8	586.1916	586.1932	2.8	B[*a*]PDE-Guo III
VII (RNA)	570	33.0	570.1978	570.1983	0.9	B[*a*]PDE-Ado I

a *Adduct designations I through VII correspond to Figures 1A,C*.

b *m/z of the protonated molecule, [M + H]^+^, of the DNA and RNA adducts revealed by nucleic acid adductomics*.

c *dGuo, 2′-deoxyguanosine; Guo, guanosine; Ado, adenosine*.

**Figure 4:**
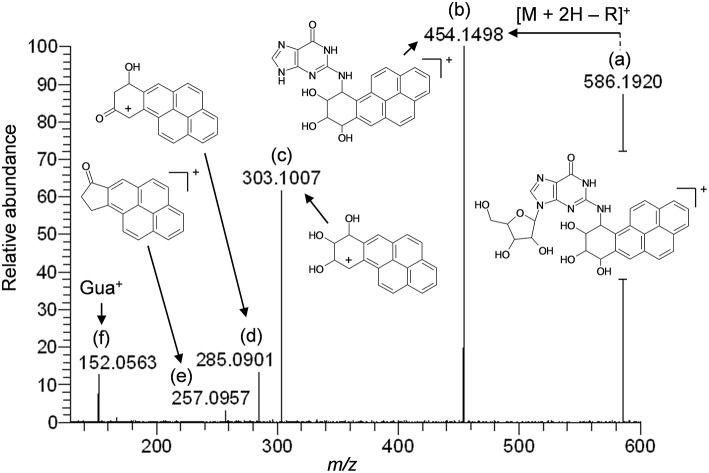
Results of LC-HRMS product ion scan analyses of putative RNA adduct V which corresponded to the protonated molecule [M + H]^+^ = 586.1920. It was targeted for product ion scan analyses after it was revealed by comparative RNA adductome analyses shown in Figure 1C. R, ribose; Gua^+^, protonated guanine. **(a–f)** mass fragments.

The results of LC-HRMS CID analyses of the most abundant putative RNA adduct, V, is shown in Figure 4. Five diagnostic product ions were detected at *m/z* 454.1498, *m/z* 303.1007, *m/z* 285.0901, *m/z* 257.0957, and *m/z* 152.0563. Compared to the fragmentation products revealed by CID analyses of DNA-derived product II fragments, these product ions occurred with identical (*m/z* 303.1007 and *m/z* 152.0563) or closely similar mass values (*m/z* 454.1498, *m/z* 285.0901, and *m/z* 257.0957; mass differences of 0.2, 0.4, and 1.2 ppm, respectively). Interpretation of the fragments shown in Figure 4 were indicative of a B[*a*]PDE-type adduct of guanosine in RNA whereby a loss of 132.0422 amu occurred from the parent protonated molecule, [M + H]^+^ = 586.1920, to produce *m/z* 454.1498, [M + 2H – R]^+^. Similarly, comparison of the observed mass value for the protonated molecule of putative RNA adduct V, [M + H]^+^ = 586.1920 and the theoretical mass value for the proposed adduct structure of putative RNA adduct V, [M + H]^+^ = 586.1932 revealed a mass error of 2.1 ppm as shown in Table 3. Overall, these results strongly supported that the identity of this putative adduct was a diastereomer of 7,8-dihydroxy-9,10-epoxy-7,8,9,10-tetrahydrobenzo[*a*]pyrene-guanosine (B[*a*]PDE-Guo) formed in the RNA of Hep G2 cells through B[*a*]PDE. Analyses of the observed mass values and theoretical mass values for these five CID fragmentation products in accordance with their proposed structures revealed that all occurred at mass errors <3.5 ppm each and supported the proposed fragment structure identities (Table 3 and Figure 4). Identically to as revealed above, the observed masses for putative RNA adducts V and VI (Table 2), provided evidence that these compounds were also diastereomers of B[*a*]PDE-Guo (mass errors <3.0 ppm each).

**Table 3:** Results of LC-HRMS CID analyses of putative DNA adduct V.

**Peak designation^^a^^**	**Observed mass fragment value, *m/z***	**Theoretical mass fragment value, *m/z***	**Mass error (ppm)**	**Molecular formula**
a	586.1920	586.1932	2.1	C_30_H_28_N_5_$O_{8}{}^{+}$
b	454.1498	454.1510	2.6	C_25_H_20_N_5_$O_{4}{}^{+}$
c	303.1007	303.1016	2.9	C_20_H_15_$O_{3}{}^{+}$
d	285.0901	285.0910	3.2	C_20_H_13_$O_{2}{}^{+}$
e	257.0957	257.0961	1.5	C_19_H_13_O^+^
f	152.0563	152.0567	2.5	C_5_H_6_N_5_O^+^

a *Peak designations correspond to Figure 4*.

RNA adductome mapping also revealed a fourth relatively abundantly-detected putative RNA adduct, VII (Figure 1), that corresponded to the protonated molecule [M + H]^+^ = 570. LC-HRMS CID analyses of this unknown compound revealed a molar mass of 570.1978 amu and two abundant product ion fragments as *m/z* 303.1010 and *m/z* 285.0903 which corresponded to the 7,8,9,10-tetrahydrobenzo[*a*]pyrene-7,8,9-triol positive ion (C_20_H_15_${\text{O}}_{3}^{+}$; theoretical = 303.1016 amu; mass error = 1.9 amu) and its dehydrated counterpart (C_20_H_13_${\text{O}}_{2}^{+}$; theoretical = 285.0910 amu; mass error = 2.5 amu), respectively and which were identical to the fragments detected from the B[*a*]PDE-dGuo and B[*a*]PDE-Guo adducts described above. Based upon the molar mass of the protonated molecule of putative adduct VII (570.1978 amu) and a molecular formula of C_30_H_28_N_5_${\text{O}}_{7}^{+}$ (theoretical = 570.1983; mass error = 0.9) in addition to identification of the two benzo[*a*]pyrene-derived mass fragments, it was concluded that this putative RNA adduct was a diastereomer of 7,8-dihydroxy-9,10-epoxy-7,8,9,10-tetrahydrobenzo[*a*]pyrene-adenosine (B[*a*]PDE-Ado) that resulted from the reaction of B[*a*]PDE with adenosine in Hep G2 RNA (Table 2).

## Discussion

In this investigation, triple quadrupole mass spectrometry comparative DNA adductome mapping of Hep G2 cells exposed to benzo[*a*]pyrene by utilizing the neutral loss targeting of the [M + H]^+^ > [M + H – 116]^+^ transition revealed three DNA adducts that were proposed to be diastereomers of B[*a*]PDE-dGuo following LC-HRMS analyses. In parallel experiments, triple quadrupole mass spectrometry comparative RNA adductome mapping of Hep G2 cells by utilizing the neutral loss targeting of the [M + H]^+^ > [M + H – 132]^+^ transition revealed three RNA adducts that were proposed to be diastereomers of B[*a*]PDE-Guo following LC-HRMS analyses. In both cases, peaks corresponding to DNA and RNA adducts were relatively well-resolved however improvements to the chromatographic conditions used herein may be useful to increase peak resolution in future analyses, e.g., utilization of a column with a smaller internal diameter and/or application of a higher mobile phase flow rate. These DNA and RNA adducts were proposed to have been formed through biotransformation of benzo[*a*]pyrene by Hep G2 cells to B[*a*]PDE stereoisomers which reacted with Hep G2 cellular DNA and RNA (Koreeda et al., 1978; Cheng et al., 1989; Ruan et al., 2006). Detection of benzo[*a*]pyrene-derived RNA adducts by RNA adductomics that were structurally analogous to DNA adducts detected by DNA adductomics provided support that RNA adductomics may be applied as a DNA adductomics DNA adduct validation technique. Comparison of DNA adductome and RNA adductome data revealed a mass difference of 16 Da between each DNA and RNA adduct and this difference represented the absence or presence of a hydroxyl group on 2′-deoxyribose in DNA or ribose in RNA, respectively.

**Figure 5:**
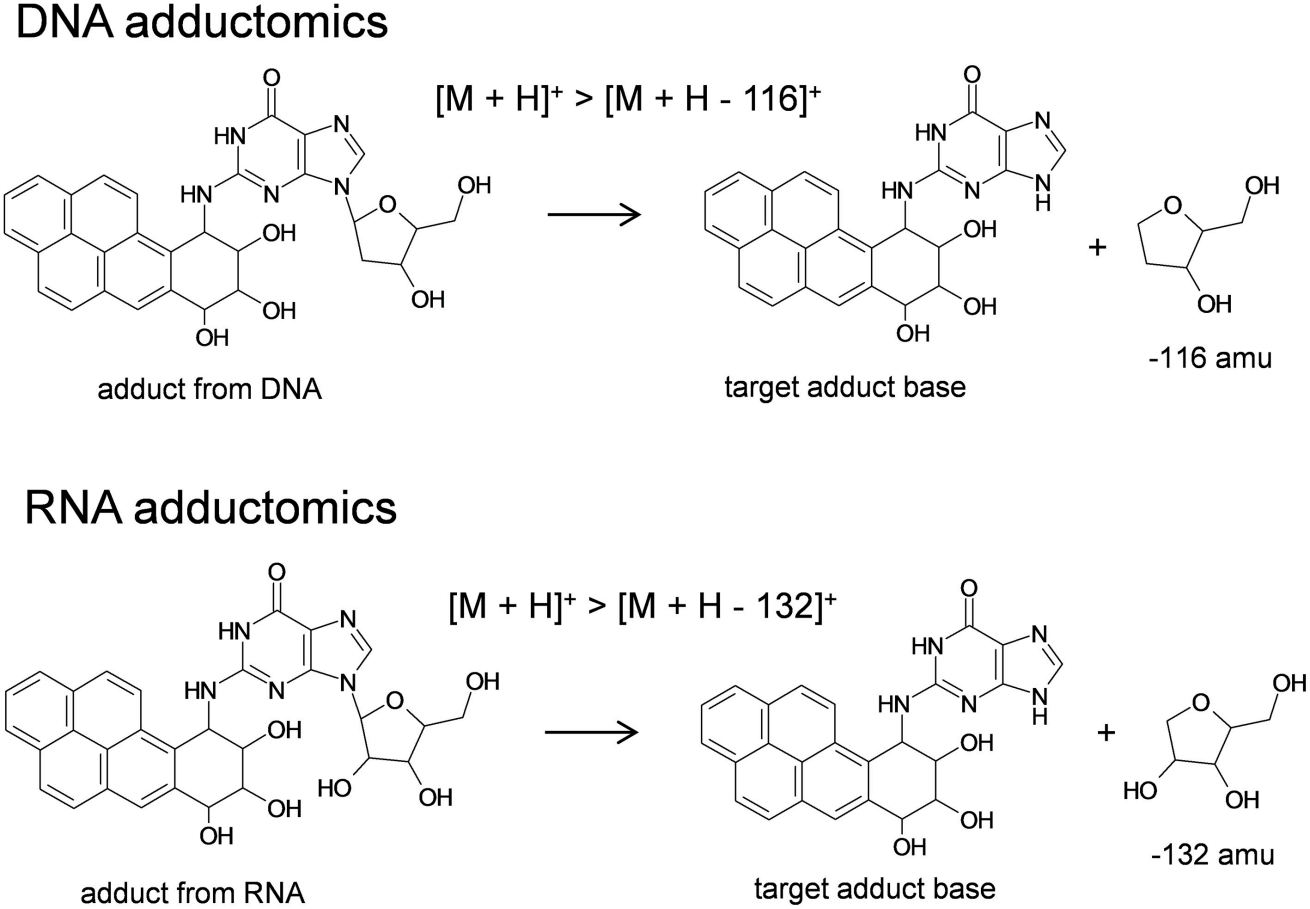
Basis for DNA/RNA adductomics analyses by LC/ESI-MS/MS.

The RNA adductomics method was based upon the understanding that ribonucleosides extracted from Hep G2 cells fragmented in the MS through their *N*-glycosidic bonds identically to 2′-deoxynucleosides and resulted in the production of protonated adducted base molecules through neutral losses of 132 amu (Figure 5). By using this approach, it was possible to design experiments to monitor [M + H]^+^ > [M + H – 132]^+^ transitions in the same manner that has been proposed for DNA adductomics. Through this process, RNA adduct analyses through RNA adductome map construction were exhibited in this work for the first time. Historically, in regard to screening for unknown RNA adducts, less research has been conducted when compared to screening for DNA adducts. This has been the case partly because RNA may not be directly involved in genotoxicity, however interest in RNA damage and the potential effects of RNA damage on cellular functioning is a topic of increasing interest (Hofer et al., 2005; Küpfer and Leumann, 2006; Zhu et al., 2006; Bullinger et al., 2007; Leung and Chan, 2015a,b; Wang et al., 2016; Wu et al., 2017). DNA and RNA possess relatively similar chemical properties and therefore they may undergo similar chemical modifications, i.e., they may be expected to exhibit similar reactivities toward nucleic acid-damaging electrophiles. It is also possible that electrophiles may display an even greater reactivity toward RNA compared to DNA. Indeed, it has been reported that DNA-damaging compounds may cause the same chemical modifications in RNA and in the few cases where research has been conducted, RNA adducts were detected in greater amounts (Hofer et al., 2005; Leung and Chan, 2015a; Wang et al., 2016). These findings point toward the possibility that RNA damage may be a more sensitive marker of genotoxicity compared to DNA, even if RNA damage is not considered to be genotoxic itself. In this work for example, a relatively abundantly-occurring putative RNA adduct (VII) was detected by RNA adductomics that was not detected in DNA adductomics and it was later identified as B[*a*]PDE-Ado. In previous work, benzo[*a*]pyrene exposure was analyzed by DNA adductomics in a CHL/IU cell line and in that case adducts of 2′-deoxyadenosine were also not detected (Kato et al., 2011).

From the perspective of *in vitro* DNA damage/genotoxicity screening, detectable DNA damage in the form of DNA adducts is often likely to occur through reaction with more nucleophilic 2′-deoxyguanosine and 2′-deoxyadenosine. The DNA/RNA adductome approach proposed herein may be useful for validation of 2′-deoxyguanosine, 2′-deoxyadenosine, and 2′-deoxycytidine adducts of DNA through detection of their structural analogs in RNA. This approach however would not be applicable to validate DNA adducts of 2′-deoxythymidine without modifications because of the presence of uridine, not 5-methyluridine—the nucleoside structural analog to 2′-deoxythymidine—in RNA. At the same time, during RNA adductome analyses, detection of artifactual DNA adducts is not likely because the loss of 132 amu from DNA adducts is not a commonly observed fragmentation mechanism. This situation may provide some safeguards against the erroneous detection of DNA adducts in RNA samples, e.g., from DNA contamination of RNA during RNA extraction.

In the context of *in vitro* genotoxicity testing overall, DNA adductomics has been discussed recently in regard to its potential usefulness to enhance chemical genotoxicity evaluations (Dertinger et al., in press). At the same time, it may also be useful for providing information in regard to mechanisms of genotoxic action (Dertinger et al., in press). Because the DNA/RNA adductome approach directly detects the potentially fundamental event of mutation initiation, i.e., nucleic acid modification of DNA, it may be useful as a complementary assay to other tests. Considering the similarities of the chemical properties of DNA and RNA, RNA adductome analyses may be the best available technique to evaluate the validity of DNA adductome results objectively. Taken from a different perspective, the detection of specific RNA adducts from RNA adductome analyses may also be useful in the evaluation of potential false negative results in DNA adductome analyses. This is the first proposal of this concept and more work shall be necessary to understand the value of applicability of DNA/RNA adductomics.

Overall, this study showed that DNA/RNA adductomics may be applied as a method for confirming putative DNA adducts that were detected by DNA adductomics. Analogous forms of nucleic acid damage were detected and identified by LC-HRMS in both DNA and RNA which supported that putative DNA adducts detected by DNA adductomics were confirmed to be DNA adducts. *In vitro* DNA adductome methods in conjunction with *in vitro* RNA adductome methods were proposed as a unique DNA adductome verification approach by which to eliminate false positive annotations if DNA adductomics is applied as a DNA damage/genotoxicity screening test. At the same time, DNA/RNA comparative adductome approaches may be used to obtain information at the molecular level in regard to the causal structure of the nucleic acid modification if needed. These types of follow-up analyses shall provide information regarding mechanism of action. The combination of DNA adductome and RNA adductome methods may prevent false positives (and illuminate the possibilities of false negatives) in regard to the DNA-damaging properties of chemical substances by detecting the existence of nucleic acid adducts from two different approaches which may result in more reliable and detailed evaluation of test data.

## Data Availability

The datasets generated for this study are available on request to the corresponding author.

## Author Contributions

TT conceived, designed, and conducted the experiments. Data was analyzed by TT and RK. TT and RK wrote the manuscript.

### Conflict of Interest Statement

The authors declare that the research was conducted in the absence of any commercial or financial relationships that could be construed as a potential conflict of interest.
