# Supplementary material for: In vitro DNA/RNA Adductomics to Confirm DNA Damage Caused by Benzo[a]pyrene in the Hep G2 Cell Line
Source: Front Chem. 2019 Jul 9;7:491. doi: 10.3389/fchem.2019.00491 (PMC6629907; doi:10.3389/fchem.2019.00491)
Supplement: Supplementary file 1 [file Data_Sheet_1.doc]

**Supplemental Material**

***In vitro* DNA/RNA adductomics to confirm DNA damage caused by benzo[*a*]pyrene in the Hep G2 cell line**

Toshihide Takeshita and Robert A. Kanaly

Department of Life and Environmental System Science, Graduate School of Nanobiosciences, Yokohama City University, 22-2 Seto, Kanazawa, Kanagawa, Yokohama, Japan, 236-0027


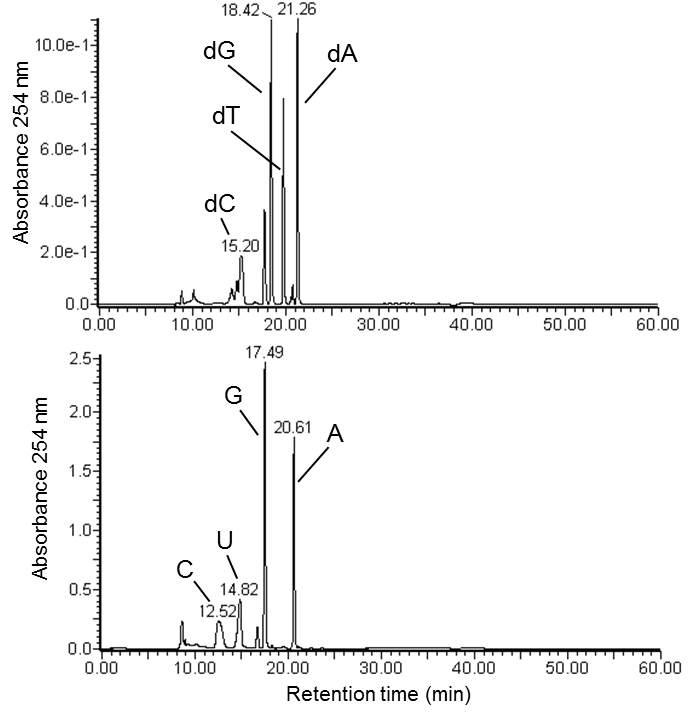


**Figure S1.** Top, UV chromatogram of digested DNA; Bottom, UV chromatogram of digested RNA.
